# Modelling of growth kinetics of *Vibrio cholerae* in presence of gold nanoparticles: effect of size and morphology

**DOI:** 10.1038/s41598-017-09357-0

**Published:** 2017-08-29

**Authors:** Tanaya Chatterjee, Barun K. Chatterjee, Pinak Chakrabarti

**Affiliations:** 1 Department of Biochemistry, Bose Institute, P1/12 CIT Scheme VIIM, Kolkata, 700054 India; 2 Department of Physics, Bose Institute, 93/1A.P.C. Road, Kolkata, 700009 India; 3 Bioinformatics Centre, Bose Institute, P1/12 CIT Scheme VIIM, Kolkata, 700054 India

## Abstract

Emergence of multiple drug resistant strains of pathogenic bacteria calls for new initiatives to combat infectious diseases. Gold nanoparticles (AuNPs), because of their non-toxic nature and size/shape dependent optical properties, offer interesting possibility. Here we report the antibacterial efficacy of AuNPs of different size and shape (AuNS10, AuNS100 and AuNR10; the number indicating the diameter in nm; *S* stands for sphere and *R* for rod) against the classical (O395) and El Tor (N16961) biotypes of *Vibrio cholerae*, the etiological agent responsible for cholera. Growth kinetics was monitored by measuring optical density at different time intervals and fitted by non-linear regression of modified Buchanan model. Sigmoidal growth curve for *Vc*O395 indicated the existence of single phenotype population and was affected by AuNR10 only, implying the importance of morphology of AuNP. Growth of *Vc*N16961 was affected by all three AuNPs indicating the vulnerability of El Tor biotype. Interestingly, *Vc*N16961 exhibited the occurrence of two phenotypic subpopulations – one with shorter (vulnerable Type 1) and the other with extended (tolerant Type 2) lag phase. Various assays were conducted to probe the impact of AuNPs on bacterial cells. Apart from AuNR10, antimicrobial efficacy of AuNS10 was better compared to AuNS100.

## Introduction

The emergence of multiple drug resistant (MDR) strains of pathogenic bacteria is a potential health threat^1^. The chemical modification of antibiotics, targeting the virulence factors has lagged behind the menace of drug resistance of many life-threatening bacteria^2^. The drug resistance is more prevalent within Gram negative bacteria, which may be due to the low permeability of the outer membrane or from the multidrug efflux pump^3, 4^. The Gram negative bacteria *Vibrio cholerae* cause the diarrheal disease cholera, which constitutes a potential threat to public health, especially in developing countries^5^. According to WHO report, more than one million people suffer from the diarrheal disease caused by *V. cholerae* infection^6^. The etiology of pathogenesis of *V. cholerae* is mainly due to O1 serogroup, which can be of two biotypes, namely, classical and El Tor. In the emerging field of nanomedicine, nanoparticles are being used to combat MDR strains^7–9^. The effectiveness of nanoparticles as antimicrobial agents is due to their high surface area to volume ratio, thereby enabling significant interactions with microbial cell membranes^10^.

Because of their unique physiochemical properties, the usage of AuNP has gained immense applications in molecular diagnostics, ranging from drug delivery to image sensing^11–13^. The non-toxic nature of AuNP also makes it an attractive candidate to combat MDR^14, 15^. Nanoparticles have specific effect on growing bacteria affecting integrity of cell membrane and thereby causing DNA damage. In fact, the variation of shape and size of AuNP is the key determinant for interaction with bacterial cells, as they attach to the bacterial cell walls prior to internalization in the cell^16–18^. In the present paper we have explored how AuNPs of different shape and size, represented by AuNS10, AuNS100 and AuNR10 (S and R being nanosphere and nanorod, respectively; 10 and 100 signify the diameter in nm) affect the growth kinetics of *Vc*O395 and *Vc*N16961. The reason for choosing AuNR is to see whether an elongation of AuNP enhances the attachment with the outer membrane surface of *V. cholerae*. Modelling of growth kinetics was done by using modified Buchanan model, which in turn gave an insight into the variation of lag phase for the biotypes. As both the biotypes were found to be vulnerable with nanorod, cell viability assay using FACS at different time intervals was carried out after treatment with AuNR. As the attachment of bacteria to the nanoparticle surface may cause damage of cell membrane, we conducted membrane fluidity assay by fluorescence anisotropy using the fluorescent probe DPH. As the membrane fluidization may lead to the protein leakage and damage of DNA, we carried out Bardford’s protein quantitation assay and fluorescence based assay using DAPI for DNA damage.

The unique feature of the existence of two phenotypic subpopulations for *Vc*N16961 compared to the classical one has been revealed by modelling and fitting the data of growth kinetics. Though two distinct phenotypes of *Vc*N16961 have been observed before^19^, to the best of our knowledge this unique feature of *V. cholerae* biotypes and their individual responses towards AuNP, based on the modelling of growth kinetics have not been reported in literature.

## Theory

### Growth kinetics of *V. cholerae* O395 and N16961 using AuNS and AuNR

The study of bacterial growth kinetics is an indispensable tool covering different areas of microbiology and has eventually gained immense importance for the understanding of the behaviour of microorganism in a given experimental condition^20^. Various mathematical models have been used to fit the kinetic data, which allows describing the behaviour of a particular bacterium under a specific condition. To date, various mathematical models have been reported in literature, such as the Gompertz, Monod, Barayani, etc^21^. Microbial growth curves were typically characterized by four-phases (*i.e*. lag, growth, stationary and decay). Initially bacterial growth starts from a zero (or near zero) till it reaches a maximum (growth phase), giving a sigmoidal nature of the growth curve^22^, after which it declines (death phase).

Buchanan presented a sigmoidal model^23^ of bacterial growth (eqn. 1) as

1 $$OD=O{D}_{F}+\frac{O{D}_{I}-O{D}_{F}}{1+\exp [(t-{t}_{m})/k]}$$

which is derivable from the Logistic model (assuming a linear relation between the OD and the number density of bacteria; (for details see Supporting Information) and can be rewritten as

2 $$OD=\tfrac{O{D}_{I}+O{F}_{F}}{2}+\tfrac{O{D}_{F}-O{F}_{I}}{2}\,\tanh (\frac{t-{t}_{m}}{2k})=a+b\,\tanh (ct-d)$$

where the parameters *OD*_*F*_ and *OD*_*I*_ are the estimates of the final and initial optical densities, *t*_*m*_ is the *delay time* where the *OD* is midway between *OD*_*F*_ and *OD*_*I*_, and *k* is a parameter which controls the maximum growth rate. The new lumped parameters, $a=\tfrac{O{D}_{I}+O{F}_{F}}{2}$, $b=\tfrac{O{D}_{F}-O{F}_{I}}{2}$, $c=\frac{1}{2k}$, and $d=\frac{{t}_{m}}{2k}$ are used here to simplify the equations. This was found to be suitable for the growth kinetics of *Vc*O395. Lag or delay times are envisaged from other growth models, however, this model allows for the delay time *t*_*m*_ to vary independent of *k*, which governs the maximum growth rate.

When modelling a bacterial population comprising of two phenotypes, the Buchanan model (eqn. 2) can be modified as

3 $$OD=a+b\,\tanh (ct-d)+e\,\tanh (ft-g)$$

where the parameters *e*, *f* and *g* have similar functions as of *b*, *c* and *d* for the *second* phenotype (here the first and second subpopulations are arbitrarily referred to as the two phenotyes). Such a co-existing two variant model has been found suitable in the growing bacterial colony of *Vc*N16961.

It may be added that the derivation of eqn. 3 for the growth of two populations implicitly ignores the interaction and/or switching between the two phenotypes as an approximation. The Buchanan model used here is based on the Logistic model. This in general, is applicable to most species of bacteria, which do not depend on co-operativity (Allee effect).

## Results

### Growth kinetics of *Vc*O395 strains in the presence of AuNR10

**Figure 1:**
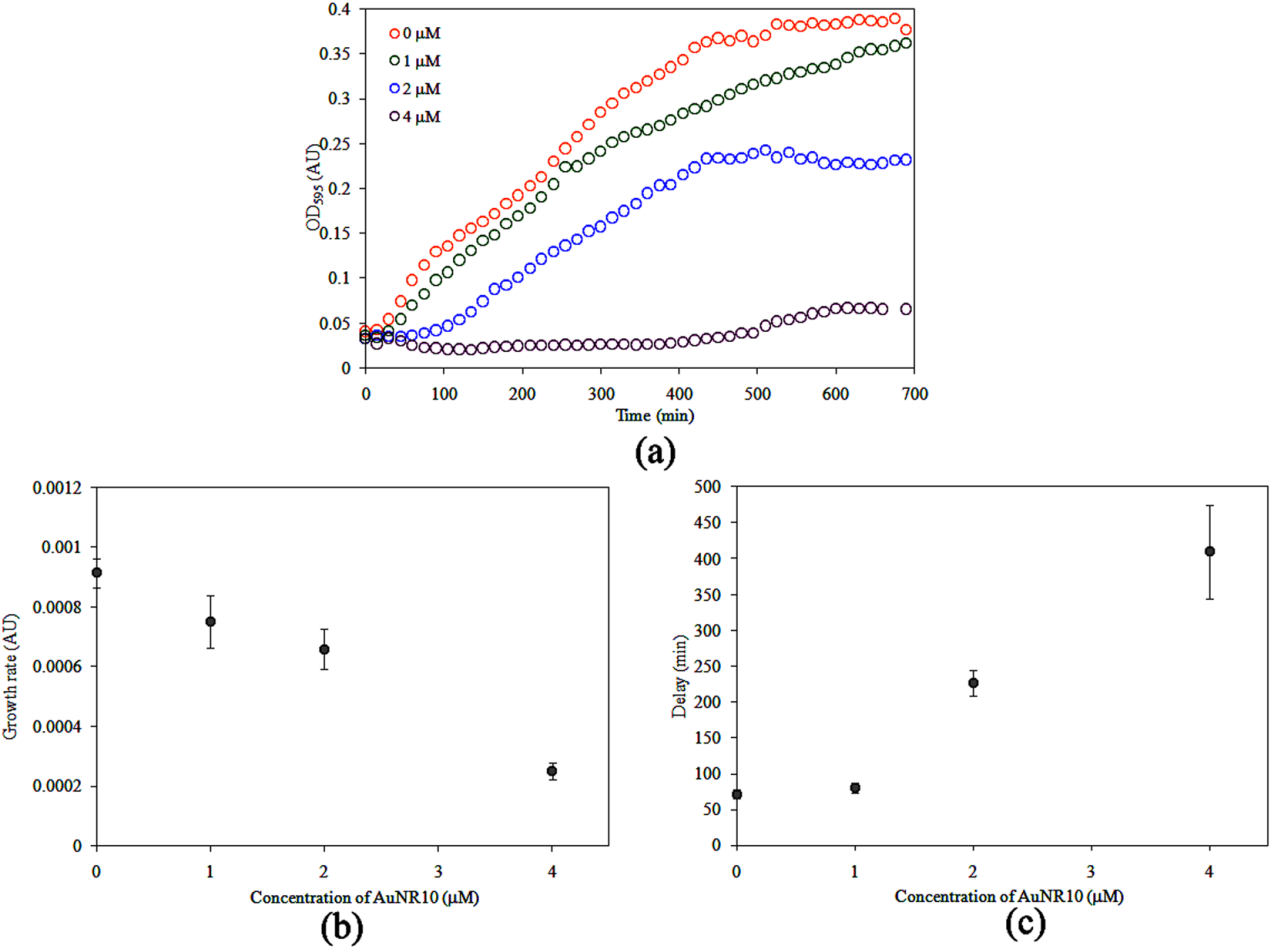
(**a**) Growth kinetics and the variation of (**b**) growth rate and (**c**) delay time of *Vc*O395 with different concentration of AuNR10.

The growth kinetics of both biotypes of *V. cholerae* was monitored by measuring OD at 595 nm at different time intervals. For *Vc*O395 strain, growth curve was found to be predominantly sigmoidal both in the absence and presence of AuNR10 (Fig. 1a). The data fitted well to the Buchanan model, as given in eqn. (2), indicating that *Vc*O395 was dominated by a single phenotype of bacteria. Also, the growth of *Vc*O395 was found to be affected only by AuNR10 and not by AuNS10 or AuNS100 (Fig. S1), implying the importance of morphology on bacterial growth^24^.

The maximum growth rate (or the maximum slope *s* of OD_595_ with time) of *Vc*O395 in the presence of AuNR10, obtained from data fitting is given by the following equation (eqn. 4) and is shown in Fig. 1b.

4 $$s=\{9.0243-1.0500[AuNR]-0.14283{[AuNR]}^{2}\}\times {10}^{-4}$$

The maximum or saturation value of the OD can be fitted by the following equation (eqn. 5)

5 $$OD=0.4247-0.019161[AuNR]-0.01699{[AuNR]}^{2}$$

which implies the antibacterial efficacy of AuNR10 for *Vc*O395. The kinetic parameters obtained for *Vc*O395 is given in Table 1. The efficiency of AuNR10 against *Vc*O395 is also reflected from the *IC*_50_ value of 3.04 μM obtained from the growth rate (Fig. 1b).

**Table 1:** Kinetic parameters obtained for *Vc* O395 using AuNR10.

Conc. of AuNR10 (μM)	Maximum OD (AU)	Delay time (min)	Maximum growth rate (× 10^−4^ OD/min)
0	0.42 ± 0.03	72 ± 7	9.1 ± 0.5
1	0.39 ± 0.04	80 ± 7	7.5 ± 0.9
2	0.31 ± 0.03	226 ± 17	6.6 ± 0.7
4	0.07 ± 0.003	410 ± 66	2.5 ± 0.3

The “lag phase”, originally described by Monod, is an important phase of bacterial growth cycle in which cells adopt to the new environment^25, 26^. This lag phase continues till the “delay time” (*t*), the time where the population of the bacteria reaches half that of maximum value. The delay time (in min) was found to increase with the increasing concentration of AuNR as seen in Fig. 1c.

This observed trend indicated that the extended time taken by *Vc*O395 was to overcome the stress created by AuNR10 before reaching the log phase. Here OD_595_ is half way between minimum and maximum values, and the slope is also a maximum.

### Growth kinetics of *Vc*N16961 strain in the presence of AuNS10 and AuNS100

**Figure 2:**
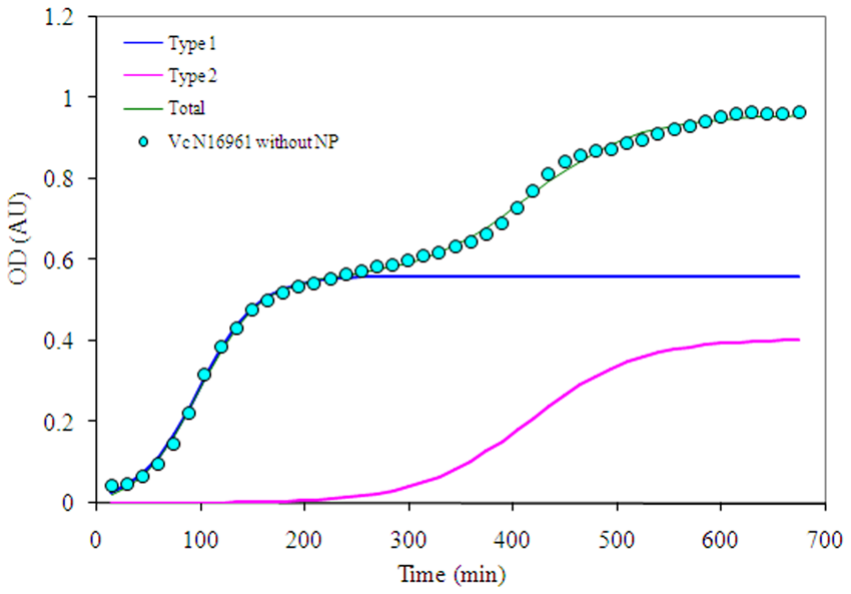
Growth kinetics of *Vc*N16961 showing the existence of two subpopulations: Type 1 and Type 2 even in the absence of AuNPs.

**Figure 3:**
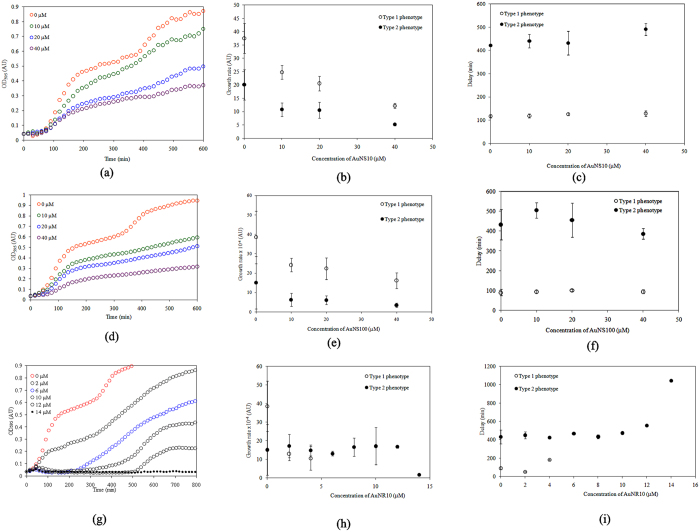
Growth kinetics, variation of growth rate and delay time of *Vc*N16961 with different concentration of AuNPs. (**a–c**) For AuNS10; (**d–f**) for AuNS100; (**g–i**) for AuNR10.

The growth kinetics of *Vc*N16961 was found to be affected by AuNP of different size (AuNS10 and AuNS100) and shape (AuNR10), indicating the vulnerability of the El Tor biotype towards AuNP over the classical one. The striking feature of the growth kinetics of *Vc*N16961 is in the existence of two phenotypic subpopulations (Type 1- with a shorter lag phase; Type 2 - with a longer lag phase) both in the absence and presence of AuNP. The growth curve of *Vc*N16961 in the absence of nanoparticles is shown in Fig. 2. Data obtained for the growth curves after treatment with AuNS10 were analyzed and fitted using eqn. (3) (Fig. 3a, Table 2).

**Table 2:** Kinetic parameters obtained for *Vc* N16961 using different AuNPs.

	Type1 (vulnerable phenotype)				Type2 (tolerant phenotype)		
	Conc. of AuNPs (μM)	Maximum OD (AU)	Delay time (min)	Maximum growth rate (x 10^−4^ OD/min)	Maximum OD (AU)	Delay time (min)	Maximum growth rate (× 10^−4^ OD/min)
Without AuNP	0	0.22 ± 0.05	90 ± 16	38.5 ± 13.5	0.17 ± 0.03	433 ± 76	15.1 ± 13.5
With AuNS10	10	0.16 ± 0.02	118 ± 9	24.7 ± 2.6	0.15 ± 0.02	441 ± 30	10.8 ± 2.6
	20	0.12 ± 0.02	126 ± 6	20.6 ± 2.7	0.12 ± 0.01	432 ± 51	10.5 ± 3.1
	40	0.11 ± 0.01	128 ± 12	12.2 ± 0.9	0.06 ± 0.01	491 ± 25	5.1 ± 0.4
With AuNS100	10	0.15 ± 0.03	95 ± 8	24.2 ± 3.4	0.17 ± 0.04	505 ± 39	6.3 ± 3.4
	20	0.11 ± 0.02	101 ± 5	22.4 ± 5.5	0.12 ± 0.03	456 ± 86	6.1 ± 2.2
	40	0.08 ± 0.01	95 ± 9	16.2 ± 4.1	0.13 ± 0.03	387 ± 28	3.5 ± 1.2
With AuNR10	2	0.09 ± 0.03	50 ± 5	13.0 ± 3.8	0.38 ± 0.10	450 ± 36	17.2 ± 6.3
	4	0.03	182 ± 9	10.6 ± 6.4	0.44	425 ± 13	14.8 ± 3.0
	6	—	—	—	0.30 ± 0.03	468 ± 1	13.1 ± 1.3
	8	—	—	—	0.26 ± 0.06 0.18 ± 0.04	435 ± 23	16.5 ± 4.8
	10	—	—	—	0.1	475 ± 16	17.1 ± 10.0
	12	—	—	—	0.02	555 ± 13	16.7 ± 0.8
	14	—	—	—		1044	1.62

The maximum growth rate (or the maximum slopes of OD_595_ of the two subpopulations with time) *s*_1_ and *s*_2_ for two subpopulations of *Vc*N16961 in presence of AuNS10 as obtained from modified Buchanan model can be represented by eqns 6 and 7 as

6 $${s}_{1}=0.00353{e}^{-0.0271[AuNS10]}$$

and

7 $${s}_{2}=0.00181{e}^{-0.0316[AuNS10]}.$$

It is evident from Fig. 3b that the growth rate constants for both the subpopulations decreased with the increase in concentration of AuNS10. The delay times for two subpopulations vary very little with concentration AuNS10 (Fig. 3c) and are given by eqns 8 and 9 as

8 $${t}_{1}=116.73{e}^{0.3114[AuNS10]}$$

9 $${t}_{2}=417.16{e}^{1.6645[AuNS10]}.$$

The *IC*_50_ (obtained from the growth rate, Fig. 3b) value for Type 1 subpopulation was found to be 21.9 μM, while that for Type 2 was 25.6 μM.

The growth kinetics of *Vc*N16961 was also studied using AuNS100 to understand the effect size of NP on the two subpopulations (Fig. 3d). The maximum growth rates (eqns 10 and 11) for two subpopulations of *Vc*N16961 in presence of AuNS100 as obtained from modified Buchanan model shows that

10 $${s}_{1}=0.0034{e}^{-0.0199[AuNS100]}$$

11 $${s}_{2}=0.0012{e}^{-0.0329[AuNS100]}$$

For AuNS100 the subpopulation of Type 1 phenotype declines faster than Type 2 phenotype with the increase in concentration of AuNS100 as evident from Fig. 3e. The delay times obtained for the Type 1 and Type 2 phenotypes (eqns. 12 and 13) with the change in concentration of AuNS100 (Fig. 3f) can be shown as follows

12 $${t}_{1}=93.282+0.119[AuNS100]$$

13 $${t}_{2}=475.44-1.7289[AuNS100]$$

The *IC*_50_ values obtained for *Vc*N16961 using AuNS100 were 21 μM and 34.8 μM respectively, for Type 1 and Type 2 (Fig. 3e). This indicates that the Type 2 subpopulation is much more tolerant to AuNS100, while the Type 1 shows the same vulnerability as that observed with AuNS10.

### Growth kinetics of *Vc*N16961 strain in the presence of AuNR10

Growth kinetics of *Vc*N16961 in presence of AuNR10 showed varied effects on the two subpopulations (Fig. 3g). Type 1 phenotype, which is marked by a shorter lag-phase, showed extreme susceptibility to AuNR10 causing it to vanish even at a low concentration of about 4 μM. Beyond 4 μM of AuNR10, modelling was done using single population model due to the survival of Type 2 phenotype (the one with the longer lag-phase) only. The maximum growth rate (eqn. 14) obtained for Type 1 phenotype of *Vc*N16961 using AuNR10 was

14 $${s}_{1}=0.0033{e}^{-0.3233[AuNR10]}$$

and for Type 2 was found to be constant at about 0.0016 ± 0.0002 OD/min up to a concentration of 12 μM, after which it declined.

The lag-phase of Type 1 phenotype increased rapidly with concentration of AuNR10, while that for Type 2 phenotype varied little up to about 10 μM after which it increased rapidly with the increasing concentration of AuNR10. This difference in behaviour of two subpopulations to the stress created by AuNR10 is perplexing. The present observation may imply that even a low concentration of AuNR10 led to selective growth of tolerant Type 2 phenotype by enabling them to multiply over the vulnerable Type 1 phenotype. The *IC*_50_ values for Type 1 phenotype was found to be as low as 2.1 μM (Fig. 3h) indicating extreme vulnerability of this phenotype of El Tor towards nanorod.

### Flow cytometric analysis of *V. cholerae* biotypes after treatment with AuNR10

To probe the effect of AuNR10 on the growth of *Vc*O395 and *Vc*N16961 strains, we performed FACS analysis at different time intervals using membrane permeable dye PI, which intercalates with the DNA of the compromised cells. The antimicrobial efficacy of AuNR10 was evident from the increase in the population of PI stained cells for both biotypes (Fig. S2, Table S1) with the increase in the incubation time with NP. The greater vulnerability of El Tor biotype compared to the classical one was again evident from the shift in cell populations from LL to UL implying higher percentage (13.8% after 1 h) of DNA damage for *Vc*N16961 compared to *Vc*O395 (18.96% after 1 h).

### Effect of AuNS10 and AuNR10 on membrane fluidity of *V. cholerae* strains

**Figure 4:**
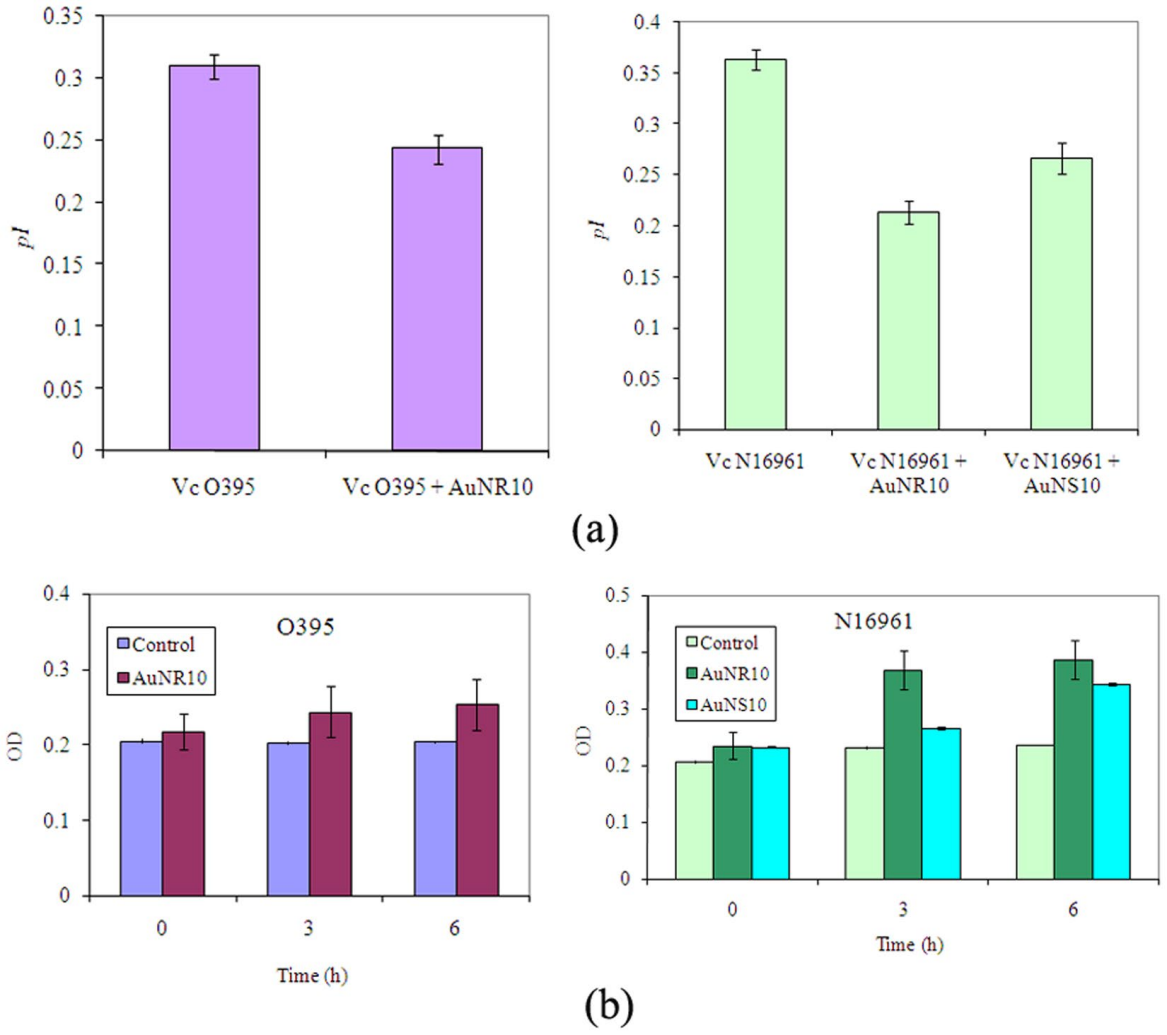
Effect of AuNPs on (**a**) membrane fluidity (*pI*) and (**b**) protein leakage of classical and El tor biotypes of *V. cholera*.

For monitoring fluidity of cell membrane the rod shaped fluorescent probe DPH is widely used reflecting their rotational motion in membrane^27^. DPH is almost non-fluorescent in aqueous environment; the fluorescent intensity, however, largely increased when DPH binds to the hydrophobic region of cell membrane, essentially composed of lipid bilayer^28^. This lipid bilayer acts as a mechanical barrier on the rotational mobility of DPH molecules. Hence “fluidization” of membrane leads to increase in mobility of the dye, as reflected from a low *pI* value, whereas “rigidity” or “integrity” corresponds to a high value of *pI*. In the present study, the membrane integrity of *Vc*O395 was examined after treatment with AuNR10 whereas that of *Vc*N16961 was studied with both AuNS10 and AuNR10. In all the cases the membrane integrity was affected, though to different extent (Fig. 4a). On treatment with AuNR10 the membrane of *Vc*N16961 was found to be affected more as compared to *Vc*O395, as reflected from the lower value of *pI* for the former as compared to the respective control cells.

### Protein leakage from *V. cholerae* strains after treatment with AuNPs

Protein leakage caused due to damage of cell membrane for AuNP treated strains of *V. cholerae* were quantified spectroscopically by Bradford assay. Increase of protein leakage was observed with the increase in incubation time for both classical and El Tor strains after treatment with AuNR10, compared to the control cells (Fig. 4b). *Vc*N16961 was more susceptible towards AuNP – protein leakage was also observed after treatment with AuNS10. This observation may be attributed due to the greater disordered structure of cell membrane for El tor biotype, coupled with the variation in gene expression^29^. ZnO nanoparticles were also found to be more disruptive towards *Vc*N16961 cell membrane, leading to more protein leaching^30^.

### DNA degradation of *V. cholerae* strains by AuNR10

**Figure 5:**
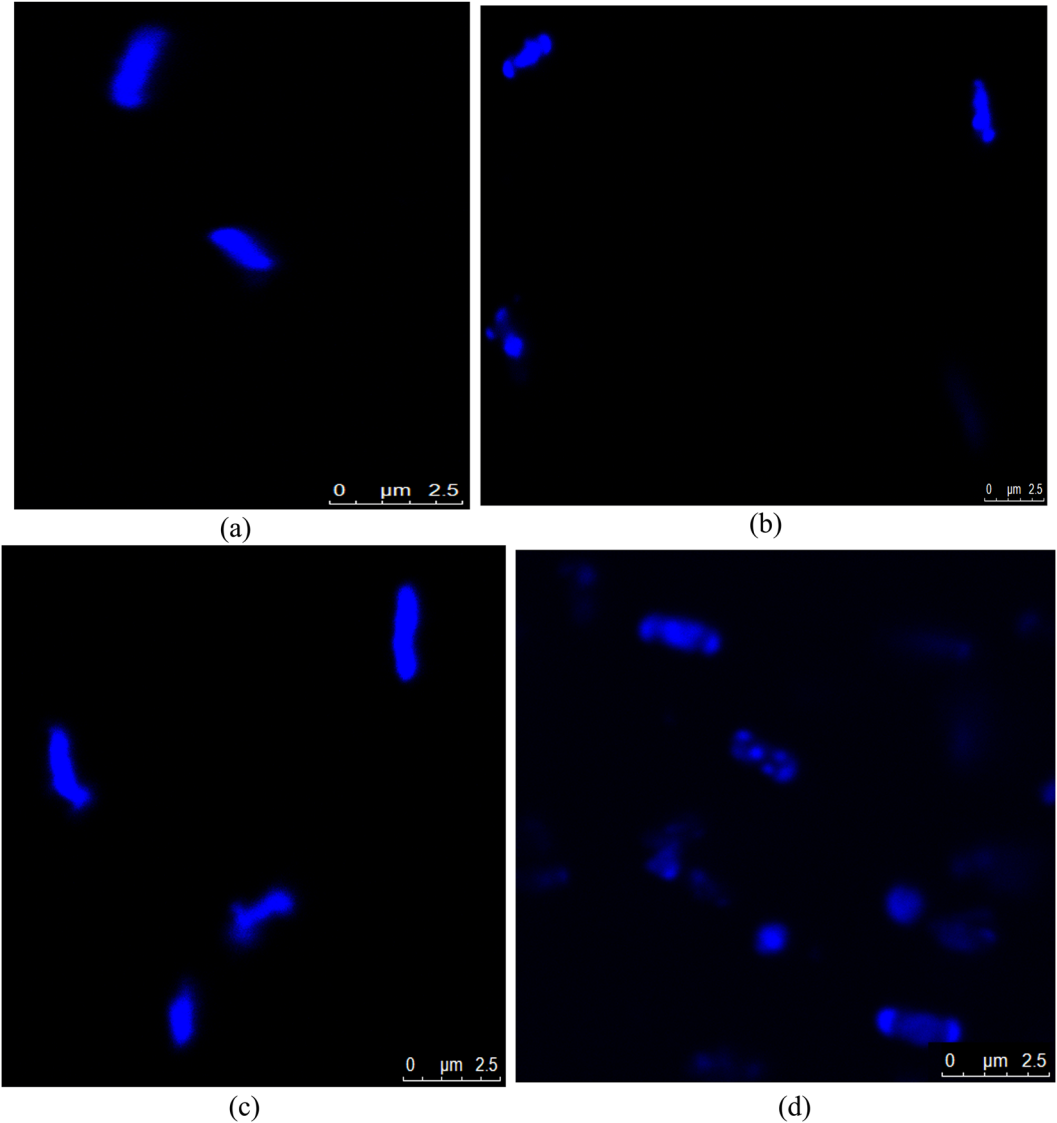
Confocal microscopic images of *Vc*O395 cells, (**a**) control and (**b**) AuNR10 treated; and of *Vc*N16961 cells, (**c**) control and (**d**) AuNR10 treated.

The impact of AuNR10 on the degradation of DNA was monitored by DAPI staining. The fluorescence dye DAPI is known to specifically bind to the minor groove of DNA with a preference for AT rich nucleic bases^31, 32^. Confocal microscope images showed fragmentation of DNA for both *Vc*O395 and *Vc*N16961 after treatment with AuNR10 (Fig. 5). This may be attributed due to the loss of integrity of cell membrane upon treatment with AuNR10.

## Discussion

The emergence of MDR strains has added a new dimension in the fight against pathogenic organisms, bolstering our search for developing alternatives to antibiotics^33^. Literature reports the potential threat of Gram negative bacteria as compared to the Gram positive one, because of the MDR exhibited by the former^3^. To date there are only a few reports of antimicrobial potency of NPs against *V. cholerae* ^33, 34^ In the present paper, we have studied the efficacy of AuNPs of different size and shape against two biotypes of *V. cholerae*, *Vc*O395 and *Vc*N16961. Out of seven cholera pandemics that the world has faced since 1817, the first six were caused by the classical one, while the El Tor biotype replaced the classical one since 1961^35^. The growth kinetics of both the biotypes revealed efficacy of AuNR10 over AuNS10. In fact, the classical one is susceptible only to AuNR10 and not to AuNS10 or AuNS100. The efficacy of AuNR10 towards *Vc*O395 is also evident from the *IC*_50_ value of 3.04 μM. There are several reports in literature stating the unique capability of nanorod in destroying pathogenic bacteria^36, 37^. AuNR10 used in our study has an aspect ratio of 3.8; an aspect ratio value of <5 is reported to have Au(100) facets on the tip of the rod and Au(110) facets on the side walls of the cylindrical rod^38^. On the other hand, AuNS10 is only composed of Au(111) facets. The higher antibacterial activity of AuNR10 compared to AuNS10 may be due to the presence of larger number of surface atoms at the corners and edges of the nanorod^39^. This may allow facile interaction of AuNR10 with lipopolysaccharides present on the outer membrane of the Gram negative *V. cholerae*, thereby allowing damage of cell membrane^40^ with the subsequent leaching of proteins present in the cytosol.

Our present study also revealed the efficacy of AuNS10 compared to AuNS100 as antibacterial agent, thereby indicating the importance of the size of NP for killing bacteria. Using AuNS10 *IC*_50_ values of 21.9 μM and 25.6 μM were obtained for Type 1 and Type 2 subpopulations of *Vc*N16961, respectively, implying greater vulnerability of Type 1. An *IC*_50_ value of 35 μM for Type 2 phenotype in presence of AuNS100 implied a greater tolerance, while Type 1 showed similar *IC*_50_ value (21.1 μM) as obtained with AuNS10.

*V. cholerae* cells are curved rod shaped having 0.36 to 0.4 μm diameter and 2.7 to 3.5 μm of length. For a spherical NP the surface area per mole is 3 *M/ρr* where *M*, *ρ* and *r* denote molar mass, density of gold and the radius of the NP, respectively. Hence smaller the radius of the NP, the larger is the surface area per mole. Considering size and steric constraints, smaller AuNS10 interacts more efficiently with bacterial cells than AuNS100 (Fig. S3). Theoretically about 85 times more AuNS10 is expected to bind to the surface of *Vc*O395 in comparison to AuNS100, considering a length 2.8 μm and radius 0.19 μm for *Vc*O395 (http://remf.dartmouth.edu/Cholera_SEM/images/03.jpg) which has a surface area of approximately 3.8 μm^2^. Hence, as the size of the NP decreases, the percentage of the surface of the *V. cholerae* covered by the nanoparticle increases, amplifying the bactericidal activity of the NP. Previous studies also reported size dependent inhibition of growth of methicillin resistant *Staphylococcus aureus* by ZnO NP of average diameter of 12 nm, while no such growth inhibition was observed with larger NP having diameter of 100 nm^24^.

The fitted data of growth kinetics after modelling was found to be strikingly different for the two biotypes. While the existence of predominantly one phenotypic subpopulation was observed for *Vc*O395, the El Tor biotype showed the distinct presence of two phenotypic subpopulations even in absence of AuNP (Fig. 2). Presence of heterogeneous subpopulations in bacterial culture has been reported in literature^40^ and this heterogeneity arises due to the difference in microenvironment^41^. A recent report by Nielson *et al*. showed the existence of two subpopulations in *V. cholerae* isolated from cholera patients using rabbit ileal loop assay for cholera^42^. The virulence genes were found to be differentially expressed on epithelial surfaces; one hyper infectious subpopulation expressing toxin co-regulated pilus (TCP) /CTX genes while the other is TCP/CTX non-expressing.

Earlier study reported the mechanism by which AuNP kills Gram negative *Escherichia coli* ^43^. The change of membrane potential of bacterial cells by AuNP led to reduction of ATP synthase activity, thereby reducing the metabolism process. In the present study the impact of AuNPs on the fluidization of cell membrane of *V. cholerae* biotypes was explored using the rotational mobility of DPH molecules. Reduction of *pI* values for NP treated cells compared to untreated cells implied fluidization of membrane and the El Tor strain was found to be more vulnerable than the classical one. As the integrity of the cell membrane is responsible for maintaining various functions of the cell, fluidization led to leakage of protein as quantified by Bradford assay (Fig. 4).

In summary, our present study revealed the unequivocal role of AuNPs as antimicrobials for targeting pathogenic *V. cholerae*. The subsequent displacement of the classical biotype by the more virulent El Tor strain since seventh cholera pandemic creates a potential threat in developing countries for combating cholera^44^. Earlier work indicated the preferential growth of *Vc*N16961 over *Vc*O395 in the small intestine^45^. The difference between two biotypes arises from the absence of twentytwo genes in *Vc*O395 compared to El Tor triggering differential virulence gene expression^29, 46^. The vulnerability of *Vc*N16961, the more virulent biotype compared to the classical one towards all types of AuNP used in this study may be of therapeutic relevance in combating secretory diarreha.

## Materials and Methods

### Materials

Gold nanospheres of different sizes (AuNS10, product code #752584 and AuNS100, product code #753688) and gold nanorod (AuNR10, product code #716812) were purchased from Sigma-Aldrich, USA and were used as supplied. LB powder was purchased from Himedia. DAPI, DPH and PI were purchased from Sigma Aldrich. All other chemicals, of analytical grade, were purchased from Merck, India.

### Bacterial strain

*V. cholerae* O395 and N16961 strains were kindly provided by Dr. R.K. Nandy (NICED, Kolkata).

### Growth kinetics of *Vc*O395 and *Vc*N16961 using AuNPs

To determine the growth curve of *Vc*O395 and *Vc*N16961 in presence of AuNPs, cells were cultured in 5 mL LB media at 37 °C overnight. Growth kinetics were performed in 96 well plates (Nunc^TM^) after adding 2% inoculum from overnight cultures in LB supplemented with different AuNPs. A control group for each strain without any AuNP was also maintained. The OD at 595 nm was monitored every 15 min in Tecan Genios microplate reader (Austria) at 37 °C with shaking for 24 h. For each strain, ten independent growth curves were monitored and the data were fitted.

### Flow cytometric analysis of *Vc*O395 and *Vc*N16961 after treatment with AuNR10

The impact of AuNR10 on the growth of both the classical and El Tor biotypes were investigated because of the vulnerability of both strains to the rod shaped AuNP. FACS of the AuNP treated cells was carried out at different time intervals using propidium iodide (PI). The unique property of PI to penetrate compromised bacterial cell membrane with concomitant binding to DNA makes it an excellent probe to distinguish between AuNP treated and untreated cells. Inoculation of fresh LB with overnight grown cultures of both biotypes was done and cells were grown to an OD of 0.4. Cells were then incubated with AuNR10 at 37 °C for different time points. After harvesting cells were washed and finally resuspended in 1X PBS. The resuspended cells were then treated with RNase A at 25 °C for 1 h. Finally the cells were incubated with 5 μl of PI in dark for 15 min. Control experiments were also carried out without AuNR10. Samples were then analyzed in a flow cytometer (FACSCalibur, BD Biosciences) equipped with a blue argon laser light source operating at 488 nm.

### Impact of AuNPs on membrane fluidity of *V. cholerae* strains

The effect of AuNS and AuNR on the membrane fluidity of *Vc*O395 and *Vc*N16961 was investigated by fluorescence anisotropy method using the fluorescent probe DPH. Cells from overnight grown cultures were added to fresh LB media (2% inoculums) and shaked at 37 °C until the OD_595_ reached 0.5. Cells were then harvested, washed with chilled 1X phosphate buffer saline (PBS) and finally resuspended in PBS to an OD_595_ value adjusted to 0.05. The cell suspension was then treated with AuNS and AuNR of desired concentration at 37 °C for 1 h. After washing and resuspension of cell pellet in 1 ml PBS, the fluorescent probe DPH was added to a final concentration of 5 μM and incubated in dark for 45 min. Finally the stained cells were washed again and resuspended in 1X PBS for fluorescence anisotropy measurement. Control experiments were conducted without nanoparticles.

The fluorescence was measured in Hitachi F-1000 spectrofluorimeter. The fluorescent probe DPH was excited at 360 nm with the vertically oriented excitation polarizer. The emitted polarized light for both vertical and horizontal planes was measured at 431 nm. During measurement each sample was stirred with a magnetic bar. The anisotropy values *pI* were measured using eqn. 15 as

15 $$pI=\frac{[{I}_{V}-{I}_{H}({I}_{HV}/{I}_{HH})]}{[{I}_{V}+{I}_{H}({I}_{HV}/{I}_{HH})]}$$

where *I* is the fluorescence emission intensities and *V* and *H* denote the vertical and horizontal orientation of the polarizer.

### Effect of AuNS and AuNR on protein leakage from bacterial cell membranes

Leakage of protein from bacterial cells was detected spectroscopically using Bradford’s reagent for protein assay. Both strains of *V. cholerae* were grown in LB at 37 °C overnight. From the overnight culture, 2% inoculums were added to LB and shaked. Cell pellet was washed and resuspended in 1X PBS, after which the cell suspension was incubated with and without AuNPs at 37 °C. Samples were withdrawn after specific time interval and were centrifuged (5000 rpm at 4 °C) for 10 min. For each sample, 100 μl of supernatant containing protein was mixed with 900 μl of Bradford reagent and incubated in dark for 10 min, after which the OD_595_ nm was measured on a Shimadzu UV Spectrophotometer UV-1800.

### Effect of AuNS and AuNR on fragmentation of DNA

To monitor DNA damage of AuNP treated strains of *V. cholerae*, cells were treated with a cell permeable stain DAPI and visualized in CLSM (Carl Zeiss, Germany). Typically, 5 ml LB was inoculated with overnight cultures of *Vc*O395 and *Vc*N16961 strains and were shaken at 37 °C for 3 h followed by 1 h treatment with AuNS and AuNR. Cell pellet was then washed with 1 X PBS buffer and incubated with 0.3 μg/ml DAPI in dark for 1 h. Excess stain was removed by washing with PBS and cell pellet was finally suspended in 1X PBS. Samples were placed on glass slide with a glass cover slip and viewed with confocal microscope using an excitation wavelength of 543 nm.

## Electronic supplementary material

Supplementary Information
