# Supplementary material for: Modelling of growth kinetics of Vibrio cholerae in presence of gold nanoparticles: effect of size and morphology
Source: Sci Rep. 2017 Aug 29;7:9671. doi: 10.1038/s41598-017-09357-0 (PMC5575114; doi:10.1038/s41598-017-09357-0)
Supplement: Supplementary file 1 — Supplementary Information [file 41598_2017_9357_MOESM1_ESM.doc]

**Modelling of growth kinetics of *Vibrio cholerae* in presence of gold nanoparticles: effect of size and morphology**

**Tanaya Chatterjee*^a^, Barun K Chatterjee*^b^ and Pinak Chakrabarti^a,c^**

^a^Department of Biochemistry, ^c^Bioinformatics Centre, Bose Institute, P1/12 CIT Scheme VIIM, Kolkata 700054, India.

^b^Department of Physics, Bose Institute, 93/1 A.P.C. Road, Kolkata 700009, India.

**Derivation of the Buchanan Model from Logistic Model and further modifications:**

The Logistic model is given as

$$\frac{dN}{dt}=rN\left(1-\frac{N}{N_{x}}\right)$$

Where *N* is the number of bacterial cells, *r* is the effective growth rate constant for Logistic model and *Nx* is the asymptotic value of *N*.

The above equation can be integrated as

$$N=\frac{N_{0}N_{x}}{N_{0}+(N_{x}-N_{0})e^{-rt}}$$

This can be rewritten as

$$N=\frac{N_{x}}{1+e^{-(t-c)/k}}$$

If one assumes, that

$$OD_{590}-OD_{F}\propto N$$

then the above equation can be written as follows representing the Buchanan Model.

$$OD_{590}-OD_{F}=\frac{OD_{I}-OD_{F}}{1+e^{(t-t_{m})/k}}$$

This can be further rearranged as

or, quite simply,

$$OD_{590}=a+b\tanh\left(c(t-t_{m})\right)$$

which was used in this manuscript.

**Table S1: Flow cytometric analysis of *V. cholerae*** biotypes at different time intervals after treatment with AuNR10

| Time (min) | *Vc*O395 | | *Vc*N16961 | |
| --- | --- | --- | --- | --- |
|  | Quad | % Gate | Quad | % Gate |
| 0 | LL  UL | 99.19  0.72 | LL  UL | 99.49  0.41 |
| 15 | LL  UL | 98.97  1.00 | LL  UL | 96.22  3.77 |
| 30 | LL  UL | 93.67  5.95 | LL  UL | 88.87  11.03 |
| 60 | LL  UL | 86.15  13.79 | LL  UL | 81.02  18.96 |

(a)

(b)

Figure S1: Growth kinetics of *Vc*O395 with different concentration of (a) AuNS10 and (b) AuNS100


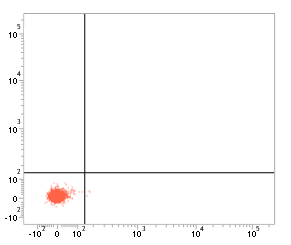

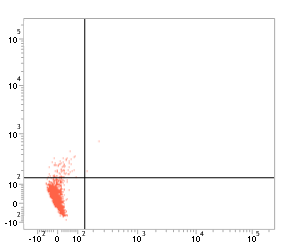


0 min 15 min


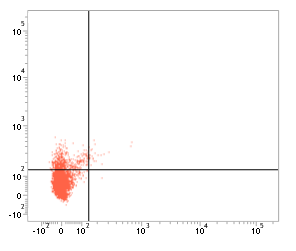

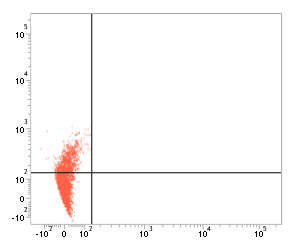


30 min 60 min

(a)


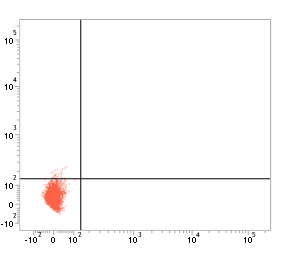

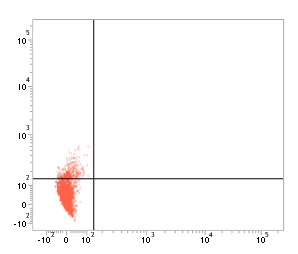


0 min 15 min


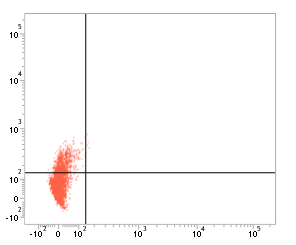

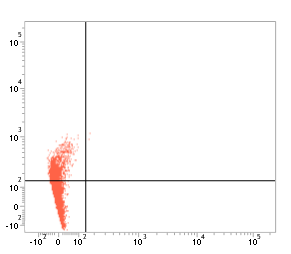


30 min 60 min

(b)

Figure S2: Flow cytometric analysis of (a) *Vc*O395 and (b) *Vc*N16961 with AuNR10 at different time intervals.


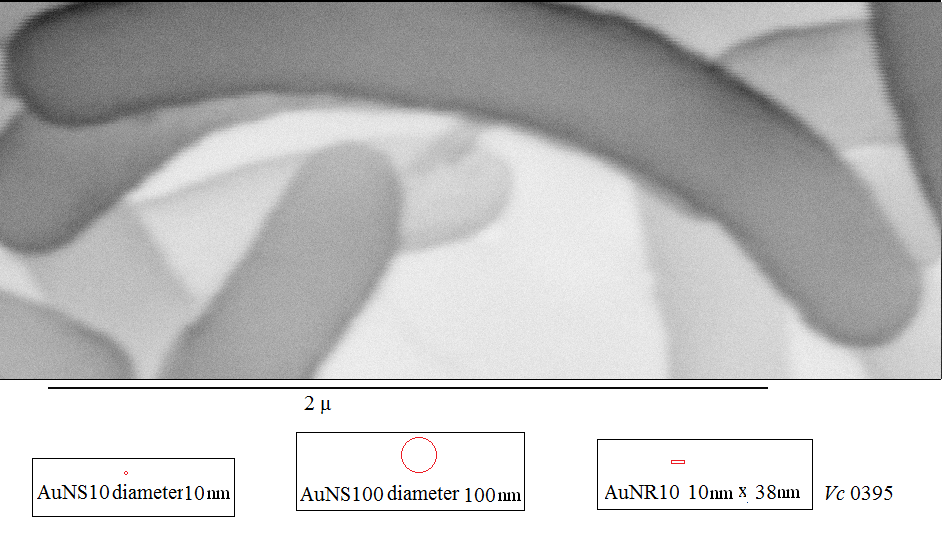


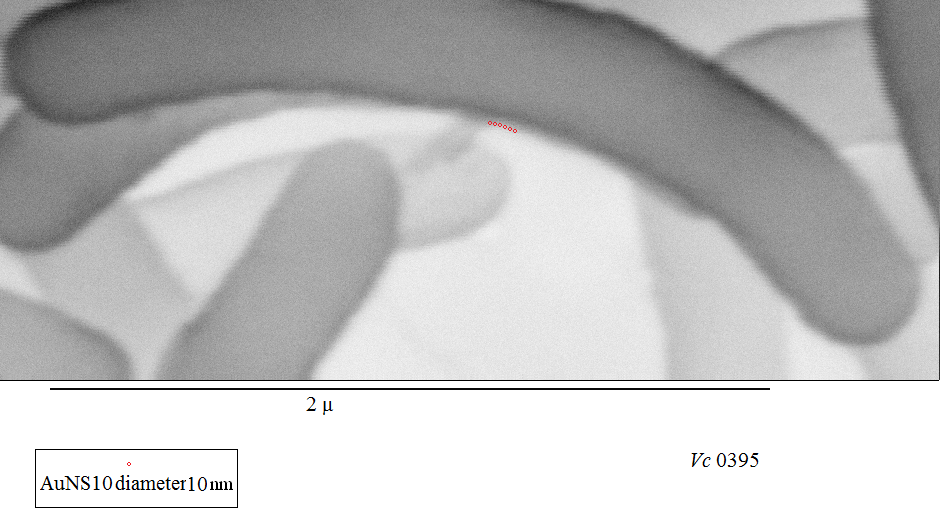


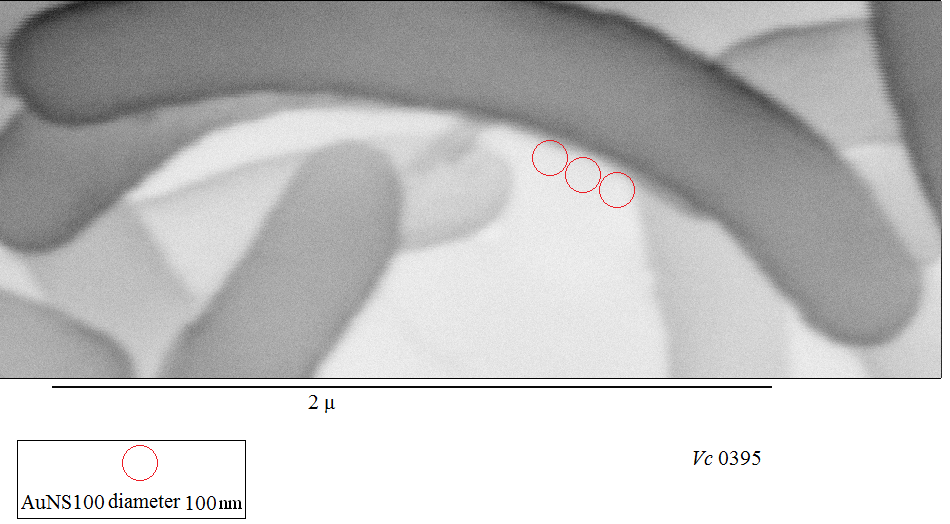


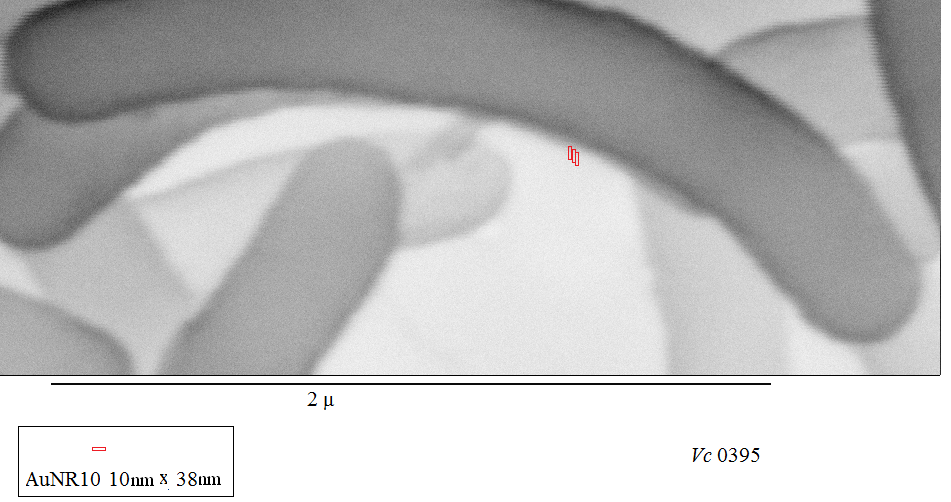


Figure S3: Schematic presentation of *Vc*O395 with different AuNPs.
